# Psychosocial and organizational barriers and facilitators of meningococcal vaccination (MenACWY) acceptance among adolescents and parents during the Covid-19 pandemic: a cross-sectional survey

**DOI:** 10.1186/s12879-022-07473-5

**Published:** 2022-05-31

**Authors:** Veja Widdershoven, Rianne P. Reijs, Amanja Verhaegh-Haasnoot, Robert A. C. Ruiter, Christian J. P. A. Hoebe

**Affiliations:** 1 Department of Sexual Health, Infectious Diseases and Environmental Health, Living Lab Public Health South, Public Health Service South Limburg, Het Overloon 2, 6411 TE Heerlen, The Netherlands; 2 Department of Social Medicine, Care and Public Health Research Institute (CAPHRI), Maastricht University, Maastricht, The Netherlands; 3 Department of Youth Health Care, Living Lab Public Health South, Public Health Service South Limburg, Heerlen, The Netherlands; 4 Department of Work & Social Psychology, Faculty of Psychology and Neuroscience, Maastricht University, Maastricht, The Netherlands; 5 Department of Medical Microbiology, Care and Public Health Research Institute (CAPHRI), Maastricht University Medical Centre, Maastricht, The Netherlands

**Keywords:** Meningococcal disease, MenACWY vaccination, Vaccination acceptance, Vaccination campaign, adolescent, parents

## Abstract

**Background:**

This study aimed to identify differences and similarities among adolescents and parents in various psychosocial factors influencing meningococcal ACWY (MenACWY) vaccination acceptance. Besides, the impact of the Covid-19 pandemic was assessed as well as resulting organizational adjustments.

**Methods:**

We conducted a cross-sectional survey among adolescents that attended the appointment for the MenACWY vaccination in South Limburg between May and June 2020, and their parents. Independent t-tests and χ^2^ test were performed to explore differences in psychosocial and organisational factors between adolescents and parents.

**Results:**

In total, 592 adolescents (20%) and 1197 parents (38%) filled out the questionnaire. Adolescents scored lower on anticipated negative affect towards MenACWY vaccination refusal [t (985.688) = − 9.32; ρ < 0.001], moral norm towards MenACWY vaccination acceptance [t (942.079) = − 10.38; ρ < 0.001] and knowledge about the MenACWY vaccination and meningococcal disease [t (1059.710) = − 11.24; ρ < 0.001]. Both adolescents and parents reported a social norm favouring accepting childhood vaccinations, but adolescent scored higher [t (1122.846) = 23.10; ρ < 0.001]. The Covid-19 pandemic did barely influence the decision to accept the MenACWY vaccination. Only 6% of the participants indicated that Covid-19 influenced their decision. In addition, the individual vaccination appointment was rated very positive. Most adolescents (71.5%) and parents (80.6%) prefer future vaccinations to be offered individually rather than having mass vaccinations sessions.

**Conclusions:**

This study provides an indication of which psychosocial and organisational factors should be addressed in future MenACWY vaccination campaigns. Individual vaccination appointments for adolescents should be considered, taking the costs and logistical barriers into account.

## Introduction

Following an outbreak of meningococcal disease caused by serogroup W (MenW:cc11) between 2015 and 2018 [1, 2], MenACWY vaccination was offered to just over one million 14 to 18 year-olds in 2018 and 2019 in the Netherlands. This resulted in 865.000 vaccinated adolescents (86%), 84% within the vaccination campaign and 2% outside the campaign [3, 4]. Since 2020, adolescents aged 14 years old have been offered MenACWY vaccination in the standard Dutch National Immunization Programme (NIP) [3, 4]. Despite the Covid-19 pandemic in 2020, MenACWY vaccination was offered with organizational adjustments in accordance with national and international guidelines.

In the Netherlands, vaccination campaigns for adolescents are normally organized group wise at public venues, such as sport centres [5]. Because of the social distancing recommendation, alternative organisation of the campaign was needed. In addition, children and parents were not allowed to come to the appointment if they had for example a mild cold or if someone in the family had a fever. These factors might have had an impact on people’s willingness to accept a vaccination.

A discussion has been initiated about whether this global experience will solve the problem of vaccine hesitancy and vaccine refusal [6]. On the one hand, people might want to avoid other disease outbreaks on top of Covid-19 and the topicality of this infectious disease threat might strengthen people’s experienced need for vaccinations preventing other infectious diseases. On the other hand, Covid-19 might be a reason to refuse or delay vaccines because of the fear of getting infected by contacts with others during the vaccination process. The pandemic might thus disrupt ongoing health care programs and the delivery of important health services, including vaccination campaigns [7].

In addition to these Covid-19 related factors, vaccination acceptance is affected by many other aspects and remains a complex phenomenon. Whether people decide to refuse, accept or delay vaccinations, involves multiple contextual, vaccine-specific, and psychosocial factors [5, 8]. Contextual factors include factors such as socio-economic status, policies and geographic barriers. Vaccine-specific factors include factors such as mode of administration and vaccination schedule. Psychosocial factors include influences arising from personal perception or influences of the social environment, such as past experiences and perceived social norm [5, 8]. Examining public attitudes about the vaccine and the targeted disease can contribute to achieving high coverage of a new vaccine [9]. Studies help us to understand motivations, facilitators and barriers that affect vaccine acceptance among different population groups [9].

The most common reasons for accepting or refusing the MenACWY vaccination have been indicated in previous studies. Common factors related to acceptance are accessibility, recommendations (from healthcare professionals), social responsibility, perceived risk, having enough knowledge and having a positive attitude [9–13]. Common reasons related to refusal include not receiving enough information, low perceived risk, and infrastructural barriers [12, 14]. Both parents and adolescents are often inadequately informed about the importance of vaccination during adolescence, such as the MenACWY vaccination [14]. Nowadays, more and more online resources are used to acquire information about vaccinations [14].

Studies examining the differences between adolescent and parental decision-making mostly used qualitative methods, including smaller sample sizes. These studies conclude that many adolescents adjust their beliefs and values to those of their parents when considering a vaccination [14, 15]. Studies suggest that factors influencing these decisions were mostly the same among adolescents and parents, but some indicated less knowledge of meningococcal disease among adolescents [9, 15, 16]. To provide tailored information and inform future campaigns we need to quantitatively examine whether beliefs and other related factors, such as organizational barriers, are different between adolescents and parents and which factors influence the decision-making in both groups.

In this study, we first identify differences and similarities in various psychosocial factors influencing vaccination acceptance (e.g. attitude, knowledge and barriers) among both adolescents (aged 14 years) and parents. Second, we examine the possible impact of the Covid-19 pandemic on vaccination attitude and acceptance in both groups. Third, we study how adolescents and their parents experienced the newly introduced organizational aspects of the MenACWY vaccination due to ruling Covid-19 regulations.

## Methods

### Study design

We performed a cross-sectional questionnaire study among adolescents (born in 2006) who attended the appointment for the MenACWY vaccination between May and June 2020, and their parents. Because of the necessity of the MenACWY vaccination, public health services in the Netherlands have ensured that the vaccination continued in an appropriate and Covid-19 adapted way. Alternative to a mass event, adolescents received an invitation for a specific time to receive the MenACWY vaccination, with five minutes between appointments.

About 3000 adolescents and their parents, living in South Limburg received an invitation to fill out the online questionnaire. Invitations with information about the study were sent by e-mail or text message. Participants were assured of their privacy and the confidential handling of their answers. Completing the questionnaire was voluntary and based on informed consent. Parents needed to give informed consent for themselves and for their child. After the invitation, two reminders were sent. The study was approved by the medical ethical committee of Maastricht University Medical Centre in Maastricht, the Netherlands (METC 2020-2261).

**Fig. 1:**
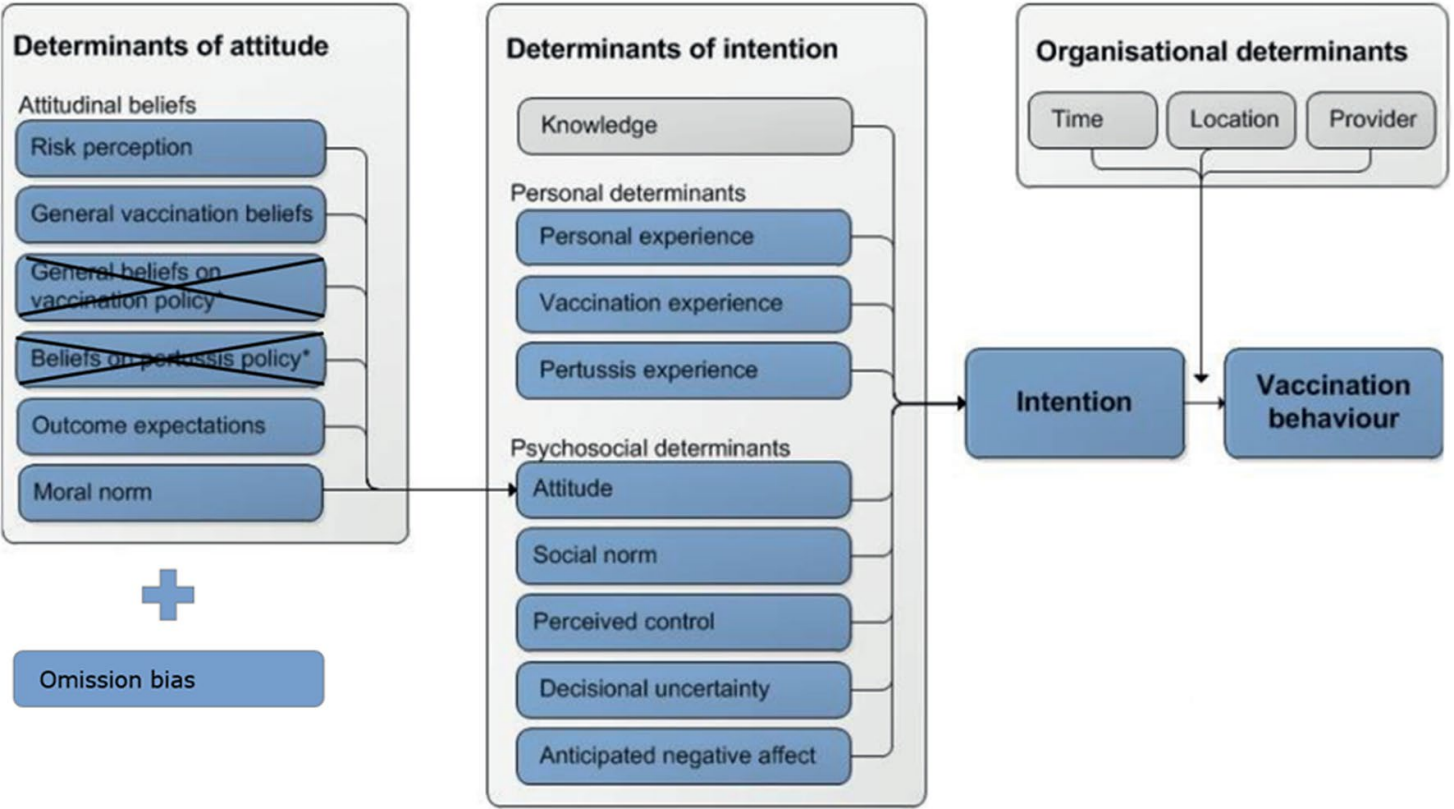
Theoretical model of Visser [17] including the changes we made

The questionnaires were based on a theoretical framework developed by Visser et al. [17]. We removed two determinants that especially play a role among healthcare professionals and added a measurement of omission bias (Fig. 1). The questionnaires comprised items measuring psychosocial (including attitudinal), personal and organizational factors as well as the influence of Covid-19 and satisfaction vaccination appointment.

Respectively seven and eight items in the questionnaire for adolescents and parents addressed the influence of the Covid-19 pandemic on their decision to receive the MenACWY vaccination, importance of vaccinations due to the coronavirus, fear of infection, willingness to get other vaccinations and whether they talked about their decision with others.

Additionally, participants were asked to evaluate their vaccination appointment for the MenACWY vaccination. This part of the questionnaire consisted of three multiple choice questions and two open questions focusing on the benefits of individual and mass vaccination. Parents only received these questions if they indicated that they had been present at the vaccination appointment. The questionnaire for the parents included overall the same constructs as the questionnaire for the adolescents. The parents’ questionnaire included more demographic variables, such as employment status.

Factors were measured with 5-point Likert scales with end-points labelled as *1 = totally disagree* and *5 = totally agree*, unless otherwise indicated. In case of sufficient internal consistency (Cronbach’s Alpha α > 0.60 or Pearson correlation r > 0.50), items were combined into one single concept.

### Statistical analysis

Frequencies were performed on sociodemographic characteristics. The data consisted of a number of child–parent dyads and therefore we performed multivariate analysis of variance (MANOVA) to check independence of both study samples. The MANOVA results showed no effect of the dyads and thus the data from parents and adolescents were treated as independent. T-tests and χ^2^ test (adequate knowledge) were used to explore statistical differences in factors between adolescents and parents. Significance was set at ρ < 0.05. Hedges’ g effect sizes were calculated to determine the magnitude of the differences between adolescents and parents. Differences between 0.2 and < 0.5 were interpreted as a small effect, between 0.5 and < 0.8 as moderate, and ≥ 0.8 as large [18]. Questions about immunisation services were analysed using descriptive analyses. SPSS statistical software (IMB, Armonk, USA, version 26) was used to analyse the data.

## Results

### Study population

The MenACWY vaccination campaign in South Limburg led to a vaccination coverage of 79%, 3999 out of 5072 adolescents accepted the vaccination in May and June 2020. We were able to approach 2943 adolescents and 3186 parents. In total, 592 adolescents (response rate 20%) and 1197 parents (response rate 38%) filled out the questionnaire. Of the adolescents who returned the questionnaire, 318 (53.7%) were girls. Most adolescents were of Dutch origin (96.3%), had a mother and father of Dutch origin (respectively 88.5% and 91.7%) and were enrolled in high level education (69.6%). The majority of adolescents received previous DTP and MMR vaccinations at the age of 9 years (n = 558, 94.3%), 133 (22.5%) adolescents received a travel vaccination and 84% of the girls received the HPV vaccination. Only a small number of adolescents (9.5%) knew someone with meningitis.

Of the parents, the majority were mothers (85.4%). Nearly half of the parents were between the age of 36 and 45 years (49.3%) or between the age of 46 and 55 years (44.9%). In most cases both parents were employed (97.6%) and of Dutch origin. Almost half of the mothers and fathers had a high educational level (respectively 48.1% and 47.0%) meaning at least higher professional education. The majority of the parents accepted the vaccinations offered to their child in the NIP (98.8%) and 17.3% of the parents indicated that their child had received a travel vaccination. Almost one third of the parents (28.3%) knew someone who suffered from meningitis.

### Psychosocial factors

Table 1 provides an overview of the psychosocial, attitudinal and organizational factors measured in this study and their internal consistency. Independent t-tests showed no differences between adolescents and parents on attitude, decisional uncertainty, moral norm for others, omission bias, and general beliefs (Table 2). Parents as well as adolescent indicated positive attitude towards the MenACWY vaccination, were satisfied with the decision to accept the vaccination, believed that vaccination is important to protect others, and reported positive beliefs about vaccination in general.

#### Perceived control, risk perception and outcome expectations

We established small differences on perceived control, risk perception and outcome expectations. Both parents and adolescents reported a positive score towards perceived control. Both feel that it is their own choice to accept a vaccine (autonomy) and that they had enough information to make an appropriate decision (capacity). Adolescents scored slightly lower on both items: perceived capacity [*t* (975.676) = − 5.09; ρ < 0.001] and perceived autonomy [*t* (1036.151)= − 7.22; ρ < 0.001]. Even though adolescents scored somewhat higher on outcome expectations [*t* (1074.312) = 6.13; ρ < 0.001], both parents and adolescents believe that vaccination will lead to a positive outcome.

In general, participants shared the belief that meningitis had a high severity, but scored lower on susceptibility of the disease and side effects. Meaning that they worry less about the spread of the disease and the side effects of the vaccine. Adolescents scored slightly lower on all three factors of risk perception: susceptibility of side effects [*t* (1055.838) = − 5.39; ρ < 0.001], susceptibility of disease [*t* (1080.602) = − 6.59; ρ < 0.001], and disease severity [*t* (1033.177) = − 8.42; ρ < 0.001].

#### Anticipated negative affect, moral norm and knowledge

We assessed moderate differences on anticipated negative affect, moral norm for oneself, and knowledge. Adolescents scored lower on anticipated negative affect [*t* (985.688) = − 9.32; ρ < 0.001] and moral norm [*t* (942.079) = − 10.38; ρ < 0.001]. However, high scores among both groups indicated that they expected to experience regret if not accepting the meningococcal vaccination, and that they feel they are expected to accept a vaccination for themselves (adolescents) or for their child (parents).

Also on total knowledge, adolescents scored lower compared to parents [*t* (1059.710) = − 11.24; ρ < 0.001]. When looking at knowledge adequacy (dichotomous), 320 (54.1%) adolescents and 946 (79.0%) parents had adequate knowledge (meaning a knowledge score of ≥ 4). Differences in adequate knowledge between adolescents and parents were significant (χ^2^ test; ρ < 0.001).

#### Social norm

Both groups reported that the social norm towards accepting the MenACWY vaccination was positive. Adolescents reported more positive social norms compared to parents [*t* (1122.846) = 23.10; ρ < 0.001], which indicates that the feeling that important others, such as friends, are positive about vaccination in general is greater among adolescents.

**Table 1:** Overview of psychosocial, attitudinal and organizational factors measured with the questionnaires

Factors	Example question Totally disagree–totally agree (scale 1–5)	Items	Alpha/Pearson
**Psychosocial factors**			
Attitude	To vaccinate against meningococcal disease, I think is (Very unimportant–very important)	Adolescents: 3 Parents: 3	α = 0.926 α = 0.965
Social norm	Friends recommend vaccinating against meningococcal disease	Adolescents: 5 Parents: 7	α = 0.789 α = 0.852
Knowledge	Meningococcal disease is caused by a bacteria (True/False)	5 items	N/A
Perceived capacity	We had enough information to make an informed decision about the MenACWY vaccination	1 item	N/A
Perceived autonomy	It is our own choice to receive a vaccination	1 item	N/A
Decisional uncertainty	The decision to accept the MenACWY vaccination feels good	Adolescents: 2 Parents: 2	r = 0.401^a^ r = 0.698
Anticipated negative affect	I would be concerned if I did not accept the MenACWY vaccination	Adolescents: 3 Parents: 3	α = 0.827 α = 0.886
**Attitudinal factors**			
General beliefs	Accepting a vaccination is self-evident	Adolescents: 8 Parents: 8	α = 0.748 α = 0.873
Moral norm (for oneself)	I feel obliged to vaccinate against meningococcal disease	1 item	N/A
Moral norm (for others)	If I would get vaccinated against meningococcal disease, I would do it to protect my environment	1 item	N/A
Perceived severity disease	Meningococcal disease is a severe disease	1 item	N/A
Perceived susceptibility disease	I am concerned that I will get meningococcal disease	1 item	N/A
Perceived severity side effects	I fear the side effects of the MenACWY vaccination	1 item	N/A
Omission bias	I prefer to get meningococcal disease, instead of getting vaccinated against meningococcal disease and risking the side effects	1 item	N/A
Outcome expectations	Accepting the MenACWY vaccination is good for my health	Adolescents: 3 Parents: 3	α = 0.773 α = 0.751
**Organizational factors**			
Organization (time)	The planned appointment suited me well	1 item	N/A
Organization (location)	The location was easy accessible	1 item	N/A
Organization (provider)	Public health services are a good place to receive vaccinations	1 item	N/A

^a^Despite a lower r among adolescents (r = 0.401), question are merged to 1 item because the r among parents was high enough (r = 0.698)

**Table 2:** Means and SD of attitudinal and psychosocial factors between adolescents and parents

Factors	Adolescents (n = 592) M ± SD	Parents (n = 1197) M ± SD	t-value	ρ -value	Effect size (Hedges’ g)
Attitude	4.37 ± 0.610	4.41 ± 0.576	− 1.34	0.179	− 0.07
Social norm	4.15 ± 0.582	3.49 ± 0.552	23.10	0.000	1.17
Perceived control					
Perceived capacity	3.66 ± 1.045	3.91 ± 0.836	− 5.09	0.000	− 0.27
Perceived autonomy	3.65 ± 1.087	4.03 ± 0.937	− 7.22	0.000	− 0.38
Decisional uncertainty	4.28 ± 0.709	4.22 ± 0.586	1.84	0.066	0.09
Anticipated negative affect	3.26 ± 0.986	3.70 ± 0.799	− 9.32	0.000	− 0.51
Knowledge	3.54 ± 1.009	4.08 ± 0.893	− 11.24	0.000	− 0.58
General vaccination beliefs	4.08 ± 0.561	3.98 ± 0.593	3.19	0.001	0.17
Risk perception					
Severity disease	3.91 ± 0.852	4.25 ± 0.732	− 8.42	0.000	− 0.44
Susceptibility disease	2.34 ± 1.057	2.68 ± 0.958	− 6.59	0.000	− 0.34
Susceptibility side effects	2.08 ± 0.968	2.33 ± 0.854	− 5.39	0.000	− 0.28
Outcome expectations	3.98 ± 0.717	3.76 ± 0.645	6.13	0.000	0.33
Moral norm					
For oneself	3.17 ± 1.195	3.75 ± 0.912	− 10.38	0.000	− 0.57
For others	3.26 ± 1.071	3.33 ± 1.003	− 1.47	0.142	− 0.07
Omission bias	1.31 ± 0.668	1.37 ± 0.613	− 1.74	0.083	− 0.09

*M *mean, *SD *standard deviation

### Covid-19 related factors

Table 3 shows that the Covid-19 pandemic barely influenced the decision to vaccinate against meningitis among adolescents and parents. Only 6% of the participants (109/1789) indicated that the pandemic influenced their decision. We assessed small differences between adolescents and parents on three Covid-19 related factors: influence decision, importance vaccination and fear contamination. First, adolescents reported a slightly higher influence of the Covid-19 pandemic on their decision to vaccinate [*t* (972.190) = 5.32; ρ < 0.001]. Second, adolescents reported a higher importance of vaccinations, as a consequence of the Covid-19 pandemic [*t* (1356.586) = 4.68; ρ < 0.001]. Last, adolescents were less afraid of infection during the appointment [*t* (1286.155) = − 6.99; ρ < 0.001]. We determined that both parents and adolescents agreed on accepting other vaccinations during the pandemic.

**Table 3:** Means and SD of Covid-19 related factors between adolescents and parents

Factors	Adolescents (n = 592) M ± SD	Parents (n = 1197) M ± SD	t-value	ρ-value	Effect size (Hedges’ g)
Covid-19 influence decision	1.90 ± 1.140	1.62 ± 0.908	5.32	0.008	0.28
Covid-19 importance vaccination	3.02 ± 1.087	2.75 ± 1.273	4.68	0.000	0.22
Covid-19 fear contamination	1.54 ± 0.877	1.86 ± 0.968	− 6.99	0.000	− 0.34
Covid-19 other vaccinations	3.95 ± 1.138	4.03 ± 1.079	− 1.48	0.139	− 0.07

*M *mean, *SD *standard deviation

### Organizational factors

Both adolescents and parents were highly positive about the accessibility, time, and provider of the vaccination (Table 4). Of the adolescents, 12.3% thought the location was not easily accessible, 10.3% was not satisfied with the time of the appointment and 2.0% thinks that vaccinations should not be provided by the public health services. Of the parents, 10.2% thought the location was not easily accessible, 12.9% was not satisfied with the time of the appointment and 2.9% thinks that vaccinations should not be provided by the public health services. Results show differences between adolescent and parents on the organizational factors time (ρ = 0.006) and provider (ρ = 0.041). However, effect size calculations indicated that these differences are trivial.

**Table 4:** Means and SD of organizational factors between adolescents and parents

Factors	Adolescents (n = 592) M ± SD	Parents (n = 1197) M ± SD	t-value	ρ-value	Effect size (Hedges’ g)
Location	3.88 ± 1.105	3.97 ± 1.077	− 1.74	0.082	− 0.08
Time	3.90 ± 1.056	3.75 ± 1.099	2.74	0.006	0.14
Provider	4.32 ± 0.787	4.24 ± 0.816	2.05	0.041	0.08

*M *mean, *SD *standard deviation

The individual vaccination appointment for the MenACWY vaccination was rated very positive; 86.3% of the adolescents and 96.2% of the parents (n = 835) thought the appointment was well organized. A total of 77.9% of the adolescents and 84.3% of the parents preferred this individual appointment compared to other forms of vaccination campaigns and 71.5% of the adolescents and 80.6% of the parents want future vaccinations to be offered individually instead of mass vaccinations. Less waiting time, less people, less fear, more personal attention and better organised are a number of frequently mentioned advantages of individual vaccination appointments (Table 5). Efficiency, choice in time, seeing friends, benefits for the provider and receiving emotional support are mentioned as advantages of mass vaccinations. However, advantages of mass vaccination were mentioned less often.

**Table 5:** Number of times an advantage was mentioned by participants

Individual vaccination appointment	Mass vaccination
Less waiting time (514)	Faster (80)
Less people (482)	Efficiency (61)
Less fear/less tension (390)	Choice of time (53)
Personal attention (231)	Seeing friends/peers (50)
Better organised (105)	Benefits for provider (45)
More privacy (86)	Receiving support (40)
Accessibility location (82)	Accessibility location (22)
Covid-19 measures (e.g. hygiene) (33)	Costs (15)

## Discussion

This study provides insights into differences in factors related to MenACWY vaccine acceptance between adolescents and their parents. Future interventions should target both adolescents and parents, in order to improve knowledge and risk perception. Moreover, this study suggested little influence of the Covid-19 pandemic on decision-making among MenACWY vaccine acceptors and their parents. Additionally, results indicated that adolescents and parents prefer individual vaccination appointments over mass vaccinations, because of less waiting time, less tension, and more personal attention.

### Psychosocial factors

In this study, we reported multiple differences between adolescents and parents on psychosocial and attitudinal factors. Adolescents scored lower on risk perception and perceived control, and higher on outcome expectations. However, effect sizes indicated that these differences were small. These small, and often meaningless, differences might occur due to the large sample size [19].

However, adolescents scored lower on moral norm for oneself and anticipated negative affect. This might be explained by the fact that adolescents worry less about the spread of the meningococcal disease [11, 16]. If someone does not feel susceptible to the disease that the vaccination is preventing, refusing a vaccination might lead to less anticipated negative outcomes. Because of the success of vaccination, adolescents have little experience with vaccine-preventable diseases, which influences their perceived risk [16]. Notwithstanding the differences between adolescent and parents on risk perception, the perceived susceptibility of meningococcal disease was low among both groups. In 2020, the incidence of meningococcal W disease in the Netherlands decreased significantly [20, 21]. It is expected that this decrease was partly caused by the measures that were taken to control the spread of Covid-19, and thereby also control the spread of other infectious diseases. The declining incidence may lead to a decreased perceived risk of meningococcal disease and a decreased necessity of vaccination [22]. Therefore it remains important to communicate about and address the risks of meningococcal disease and the necessity of the MenACWY vaccination.

We also assessed lower scores on knowledge about the MenACWY vaccination and meningococcal disease among adolescents. Around half of the adolescents (54.1%) had adequate knowledge, while 79.0% of the parents had adequate knowledge. This is in line with other studies that have identified limited knowledge among adolescents and teenagers [9, 16]. High levels of knowledge among parents are associated with socio-economic status, household income and high educational level [9]. In our study, almost half of the mothers (48.1%) and fathers (47.0%) were highly educated, and in most cases, both parents were employed (97.6%), which might be an explanation for the number of parents having adequate knowledge. Most studies reported parents as the decision-makers regarding vaccination [23, 24]. Nevertheless, 40.1% of the parents in our study indicated that they made the decision together with their child, and 68% of adolescents indicate that they ask their parents for information about vaccinations. Parent–child communication about vaccination might increase knowledge about the vaccine and the disease among adolescents [11, 15], and make adolescents feel empowered about future health-related decisions [23]. Future interventions might focus on parent–child discussions and increasing parents’ confidence in discussion vaccinations with their child.

Adolescents scored significantly higher on social norm. Previous studies have reported that adolescents are more sensitive to peer influence than adults when it comes to food intake [25]. It is not clear if this is also applicable to vaccination acceptance. However, in both groups, positive social norms toward MenACWY vaccination uptake were assessed. This is in line with a previous study, indicating more positive social norms among acceptors compared to refusers or partial acceptors [26].

### Covid-19 related factors

We reported almost no influence of Covid-19 on the decision to vaccinate against meningitis. Only 6% of the participants indicated that the pandemic influenced their decision. One explanation for these results might be the fact that the study was only able to include vaccine acceptors. Most of the participants indicated that they would also accept other vaccinations during the pandemic. Another explanation might be the effective communication about the importance of the MenACWY vaccination during the pandemic. The invitation contained additional information explaining the importance of the vaccination and the measures that were taken related to Covid-19. Moreover, attention was paid to the MenACWY vaccination campaign in regional press [27].

Little influence of Covid-19 is confirmed by the difference in vaccination rates between 2019 and 2020 in South Limburg. In 2020, 79% of the 14-year-olds was vaccinated against meningitis. In 2019, the vaccination rate among the same age group was 87%. This difference of 8% can be caused by the influence of Covid-19. Also, in 2019 the MenACWY vaccination was offered at several times during the year as the adolescents in 2019 received more invitations or reminders for the vaccination, probably explaining part of this difference.

### Organizational factors

The results of this study indicated positive scores on the organisational factors time, location and provider. In general, participants did not report any perceived organisational barriers. This can be explained by the fact that the study only included vaccine acceptors. Vaccine acceptors perceive in general fewer practical barriers compared to vaccine refusers and lower perceived organisational barriers is associated with higher vaccine acceptance [26, 28]. In addition, the MenACWY vaccination was organized at Youth Health Care locations instead of public venues and adolescents received an invitation for a specific time because of the Covid-19 measures. Most participants were very positive about their appointment.

Most participants preferred the individual appointment and want future vaccinations to be offered individually rather than having mass vaccinations sessions. Adolescents and parents experienced less stress and tension during the individual appointment. Besides, less waiting time and more personal attention were mentioned as advantages. A report from the WHO indicated that mass vaccination campaigns may contribute to immunization stress-related responses (ISRR) [29]. A crowded waiting area, lack of privacy and negative communication might be environmental causes of ISRR during mass vaccination campaigns [29]. Since 2018, the Public Health Service of Groningen implemented more individual consultations [30]. The Public Health Service South Limburg might consider individualizing the vaccination appointments for children aged 9 years and older. This means that also the costs of individual appointments need to be considered. A cost-benefit analysis of the Public Health Service of Amsterdam showed that individual appointment, including calling refusers and organizing home visits, were 40% more expensive than mass vaccinations [personal communication by Public Health Service Amsterdam].

### Limitations

Some limitations of this study need to be addressed. First, despite efforts to reach vaccine refusers, only vaccine acceptors were included. Therefore, it was not possible to study differences between vaccine acceptors and refusers. Second, selection bias is not ruled out as response was lower (20%) in adolescents than in parents (38%). This might be due to the long questionnaire (20 min) [31]. Responses are comparable to other studies, but employed parents (98%), higher educated parents (48%) and mothers (85%) seem to have participated more. This might have overestimated knowledge scores and results need to be interpreted with caution for unemployed and low educated parents and fathers.

## Conclusions

While increasing vaccination coverage remains challenging, this study provides insights into which psychosocial, attitudinal and organisational factors should be addressed in future MenACWY vaccination campaigns. The results indicate that both adolescents and parents should be targeted to improve knowledge and risk perception. Individual vaccination appointments for adolescents should be considered, while taken into account the costs and logistical barriers. To further assess psychosocial, attitudinal and organisation factors, related to MenACWY vaccination decision-making, a study among vaccine refusers should be conducted.

## Data Availability

The datasets generated and/or analysed during the current study are not publicly available due to the fact that the data of this study contain potentially identifying and sensitive participant information and that publicly sharing the data would not be in accordance with participant’s consent obtained for this study but are available from the corresponding author on reasonable request.

## Abbreviations

MenACWY: Meningococcal ACWY
Covid-19: Coronavirus Disease 2019
NIP: Dutch National Immunization Programme
DTP: Diphtheria, tetanus, polio
MMR: Measles, mumps, rubella
HPV: Human Papilloma Virus
ISRR: immunization stress-related responses
WHO: World Health Organization
